# Characterization of obesity-related diseases and inflammation using single cell immunophenotyping in two different diet-induced obesity models

**DOI:** 10.1038/s41366-024-01584-6

**Published:** 2024-07-14

**Authors:** Zsófia Ruppert, Patrícia Neuperger, Bettina Rákóczi, Nikolett Gémes, Brigitta Dukay, Petra Hajdu, Mária Péter, Gábor Balogh, László Tiszlavicz, László Vígh, Zsolt Török, László G. Puskás, Gábor J. Szebeni, Melinda E. Tóth

**Affiliations:** 1 Laboratory of Molecular Stress Biology, Institute of Biochemistry, HUN-REN Biological Research Centre, Szeged, Hungary; 2 PhD School in Biology, University of Szeged, Szeged, Hungary; 3 Laboratory of Functional Genomics, Core Facility, HUN-REN Biological Research Centre, Szeged, Hungary; 4 Department of Pathology, Albert Szent-Györgyi Medical School, University of Szeged, Szeged, Hungary; 5 Department of Internal Medicine, Hematology Centre, Faculty of Medicine, University of Szeged, H6725 Szeged, Hungary

**Keywords:** Immunology, Biochemistry

## Abstract

**Background:**

Obesity is a growing problem worldwide and a major risk factor for many chronic diseases. The accumulation of adipose tissue leads to the release of significant amounts of pro-inflammatory cytokines and adipokines, resulting in a low-grade systemic inflammation. However, the mechanisms behind the development of obesity-related diseases are not fully understood. Therefore, our study aimed to investigate the pathological changes and inflammatory processes at systemic level and in individual organs in two different diet-induced mouse obesity models.

**Methods:**

Male C57BL6/J mice were fed by high-fat diet (HFD), high-fat/high-fructose diet (HFD + FR) or normal chow for 21 weeks starting at 3 months of age (*n* = 15 animals/group). Insulin resistance was tested by oral glucose tolerance test. Pathological changes were investigated on hematoxylin**–**eosin-stained liver and brown adipose tissue sections. The gene expression levels of adipokines and cytokines were analyzed by qPCR in adipose tissues, whereas serum protein concentrations were determined by multiplex immunoassays. Immunophenotyping of isolated blood, bone marrow and spleen cells was performed by single-cell mass cytometry.

**Results:**

Weight gain, glucose intolerance and hepatic steatosis were more severe in the HFD + FR group than in the control and HFD groups. This was accompanied by a higher level of systemic inflammation, as indicated by increased expression of pro-inflammatory genes in visceral white adipose tissue and by a higher serum TNFα level. In addition, immunophenotyping revealed the increase of the surface expressions of CD44 and CD69 on various cell types, such as CD8+ and CD4 + T-cells, B-cells and macrophages, in animals with obesity.

**Conclusions:**

The combination of HFD with fructose supplementation promotes more properly the symptoms of metabolic syndrome. Therefore, the combined high-fat/high-fructose nutrition can be a more suitable model of the Western diet. However, despite these differences, both models showed immunophenotypic changes that may be associated with increased risk of obesity-related cancer.

## Background

Obesity is becoming a serious global problem. According to the World Health Organization, in 2016, 39% of adults were overweight and 13% of the world’s adult population had obesity. Globally, the incidence of obesity in 2021 was almost three times higher than in 1976, leading to an increasing number of serious health problems [1]. Obesity is an important risk factor for many chronic diseases [2]. Among them, the most well-known pathological conditions are diabetes, hyperlipidemia, cardiovascular dysfunction and non-alcoholic fatty liver disease (NAFLD), which are the main symptoms of the metabolic syndrome [3–5]. Moreover, a growing body of evidence suggests that obesity is also associated with the development of cancer, particularly those types that are located in close proximity to adipose tissues [6, 7].

The exact molecular mechanisms between the incidence of obesity and the development of these pathological conditions are not completely understood, but most likely they are related to the chronic, systemic low-grade inflammation induced by the adipose tissues. In addition to energy storage, adipocytes have important endocrine and immune functions, like the production of cytokines and hormone-like molecules called adipokines. The excessive secretion of these factors by the increasing amount of adipose tissues results in the imbalanced regulation of inflammation and whole-body metabolism [8].

High-fat diet (HFD)-fed mouse is a frequently used model to study obesity, especially certain characteristics, such as hyperlipidemia [9]. However, HFD alone may not properly model the typical human nutrition as the Western-style diet is characterized by an excessive consumption of foods that are rich in both fat and sugar. Of the dietary sugars, fructose is only found naturally in fruit and honey, which limits its excessive consumption. However, most processed foods and soft drinks are sweetened with high fructose corn syrup or sucrose, resulting in a significant rise in the daily fructose consumption in the last decades. This elevated fructose intake promotes the development of metabolic syndrome, which in turn increases the risk of diabetes, cardiovascular diseases and all-cause mortality [10].

Our study aimed to characterize and compare the cellular and molecular changes that occur during obesity-induced inflammation as well as to analyze how these changes relate to the chronic pathological conditions that develop in HFD and HFD + FR mouse obesity models.

## Materials and methods

### Animals

A total of 45 male C57BL/6 mice were used, divided into three groups (15 mice/group). The requisite sample size was determined using PS Power and Sample Size Calculation software. Animals were grouped into blocks according to age and weight group to balance these variables, and then randomly divided into ND, HFD and HFD + FR groups within each block. Mice on a standard chow diet (normal diet, ND) were used as healthy controls. Animals in the second group were fed with high-fat diet (HFD group, Supplementary Table S1). In the third group, drinking water of the HFD-fed animals was replaced by a 30% fructose solution (HFD + FR group). Dietary interventions were started at three months of age of the animals and lasted for five months. Investigators could not be blinded to the group allocations during the dietary intervention and sample collection due to the different appearance of the normal and high-fat chow, as well as the markedly elevated body weight of the animals. One animal in the ND group died during the course of the dietary intervention. Additionally, another animal in the HFD + FR group was euthanized due to a significant decline in body weight. Food (either ND or HFD) and water (or fructose solution) were available ad libitum. Mice were housed in groups of two to three in the same room under controlled conditions (24 °C, 12–12 h light-dark cycle) throughout the experiment. The experiments conformed to the EU Directive 2010/63/EU and were approved by the regional Animal Research Ethics Committee of Csongrád County (Csongrád county, Hungary; project license: XVI./847/2022.). All institutional and national guidelines for the care and use of laboratory animals were followed.

### Oral glucose tolerance test

One week before the end of the diet oral glucose tolerance test (OGTT) was performed. After 14 h of fasting, blood glucose level was measured (Accu-Chek, Roche, Mannheim, Germany). This was followed by administration of 20% dextrose solution (2 g/kg) using oral gavage. Finally, blood glucose levels were measured at 30, 60 and 120 min after glucose gavage. The area under the curves (AUC) of OGTT lines was calculated.

### Serum triglyceride, total cholesterol, LDL-cholesterol and HDL-cholesterol levels

After terminal anesthesia, blood samples were collected. Sera of heavily hemolized blood specimens were excluded from the analysis. Serum triglyceride, total, LDL- and HDL-cholesterol levels were determined as described previously [11, 12]. The detailed protocol can be found in the Supplementary Materials and Methods.

### Histological evaluation of hematoxylin–eosin-stained sections

After terminal anesthesia, the liver and interscapular brown adipose tissues (iBAT) were removed. Weights of the organs were measured, and samples were fixed in 4% paraformaldehyde (dissolved in 0.1 M PBS, pH = 7.4) for histology. Number and size of lipid droplets were analyzed using the ImageJ software on hematoxylin**–**eosin-stained sections as described previously [11]. NAFLD Activity Score (NAS) was calculated by the sum of scores of steatosis (0–3), lobular inflammation (0–3) and hepatocyte ballooning (0–2). Investigators performing histological staining, lipid droplet quantification and evaluation of NAS were unaware of the group allocation.

### RNA isolation and quantitative real-time polymerase chain reaction (qPCR)

Total RNA, isolated from the liver, visceral white adipose tissue (vWAT), and iBAT samples, was used as a qPCR reaction template after converting cDNA as described previously [11, 12]. The detailed protocol can be found in the Supplementary Materials and Methods.

### CD44 immunostaining

Following terminal anesthesia, vWAT were removed, fixed in 4% formalin, and subsequently embedded in paraffin. Immunohistochemical staining was performed on 4 μm tissue sections using a BOND-MAX Immunohistochemical staining machine (Leica). The sections were incubated with rabbit anti-CD44 antibody (Abcam cat.no: ab157107; 1:1000). A labeling system (Bond Polymer Refine Detection, DS9800, Leica) containing horseradish peroxidase (HRP) conjugated goat anti-rabbit secondary antibody and DAB-3 (3′-diaminobenzidine) as the chromogen was used to detect the antigen signal. Cell nuclei were counterstained with hematoxylin. The immunostained sections were digitally scanned using a slide scanner (MiraxMidi, 3DHistech Ltd., Budapest, Hungary). Investigators performing immunohistochemical staining and evaluation were unaware of the group allocation.

### Cell preparation and mass cytometry (CyTOF) measurement

After terminal anesthesia, blood, spleen, and bone marrow cells of the femur and tibia were isolated and processed as described previously by our group [13–15]. The antibody staining of cells was performed as described previously with minor modifications [16, 17]. The acquisition of samples for cytometry by time of flight (CyTOF) was executed as described previously with minor modifications [18–20]. The detailed protocols can be found in the Supplementary Materials and Methods.

### Measurement of plasma proteins

The measurement of plasma proteins was carried out using multiplex immunoassays. Luminex MAGPIX was performed as described previously with minor modifications [11, 21], whereas Legendplex technology was executed following the manufacturer’s instructions. The detailed protocols can be found in the Supplementary Materials and Methods.

### Statistical analysis

Statistical analysis was performed using Sigmaplot 12.0 for Windows (Systat Software Inc., San Jose, CA, USA). First equal variance and the normal distribution (Shapiro–Wilk normality test) of the data was checked. In the case of equal variance and normal distribution, one-way analysis of variance (ANOVA), followed by the Bonferroni post hoc test, was performed. In those cases where the normality test failed, the Kruskal–Wallis test by ranks (i.e., ANOVA on ranks) was performed. In case of significant differences between groups, the Dunn’s Method post hoc test was used after ANOVA on ranks. qPCR data are presented as % of the control group, Student’s *t* tests were performed for pairwise comparisons. Statistical analysis of CyTOF’s data was performed using two-tailed, homoscedastic Student’s *t* test to evaluate the statistical significance (set at *p < 0.05, **p < 0.01, ***p < 0.001) using pairwise comparison of two samples in Microsoft® Excel™. The level of statistical significance was set at *p* < 0.05. All values are presented as mean ± SD.

## Results

### Assessment of obesity by measuring weight gain, insulin resistance and dyslipidemia

**Fig. 1:**
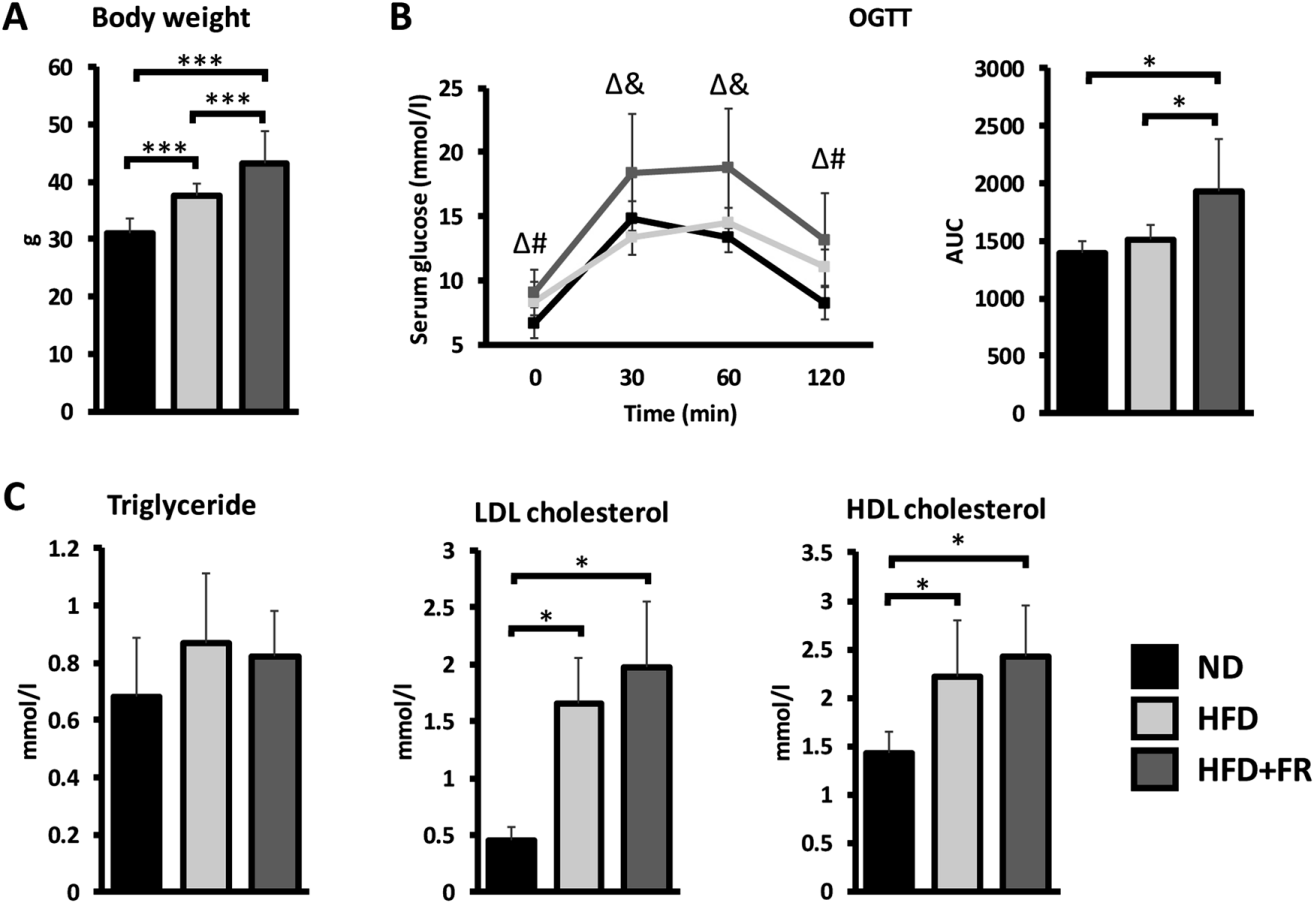
The effects of diets on body weight and serum laboratory parameters. **A** Body weight; **B** Serum glucose levels and area under the curve (AUC) values in the oral glucose tolerance test (OGTT); **C** Serum lipid profile: triglyceride, LDL-cholesterol and HDL-cholesterol levels. Values are mean ± SD, *n* = 13–15/group, ^*^p < 0.05, ^#^*p* < 0.05 ND vs. HFD groups, ^Δ^*p* < 0.05, ND vs. HFD^+^FR groups, ^&^*p* < 0.05, HFD vs. HFD + FR groups; ND normal diet, HFD high-fat diet, HFD + FR high-fat/high-fructose diet.

Body weights of HFD-fed animals significantly increased compared to the healthy animals (*p* < 0.05). However, an even higher weight gain was observed in the HFD + FR group (*p* < 0.05) leading to significant differences between the two obesity models (*p* < 0.05) (Fig. 1A). HFD alone was not enough to induce significant increase in the weights of dissected organs (liver, BAT, spleen, kidney, heart, and brain; Supplementary Fig. S1). In contrast, the weights of the liver (*p* < 0.05) and BAT (*p* < 0.05) significantly elevated in response to HFD + FR compared to the control group. Moreover, the differences between the HFD and HFD + FR animals were also significant for the liver (*p* < 0.05), BAT (*p* < 0.05), kidney (*p* < 0.05) and spleen (*p* < 0.05) (Supplementary Fig. S1).

To monitor the development of glucose intolerance in response to diets and to reveal insulin resistance (IR), OGTT was performed. We found significantly elevated fasting glucose levels in both obesity models compared to the healthy group (*p* < 0.05 for both obesity models). However, animals in the HFD + FR group showed substantially higher peak glucose levels than those in the control and HFD groups (*p* < 0.05 both at 30 and 60 min) (Fig. 1B, left). Accordingly, the area under the curve (AUC) value of the OGTT line in the HFD + FR group was significantly higher compared with the ND (*p* < 0.05) and HFD (*p* < 0.05) animals, while it did not differ between mice fed by ND and HFD (Fig. 1B, right).

To compare the severity of diet-induced dyslipidemia in the different obesity models, we measured serum triglyceride, LDL- and HDL-cholesterol levels. Serum triglyceride levels did not elevate significantly either in the HFD or in the HFD + FR groups (Fig. 1C, left). In contrast, LDL- and HDL-cholesterol levels were significantly higher in both the HFD (*p* < 0.05) and HFD + FR (*p* < 0.05) groups than in the healthy animals, while we could not detect difference between the two obesity models (Fig. 1C, middle and right).

### Diet-induced inflammation characteristics of the liver

**Fig. 2:**
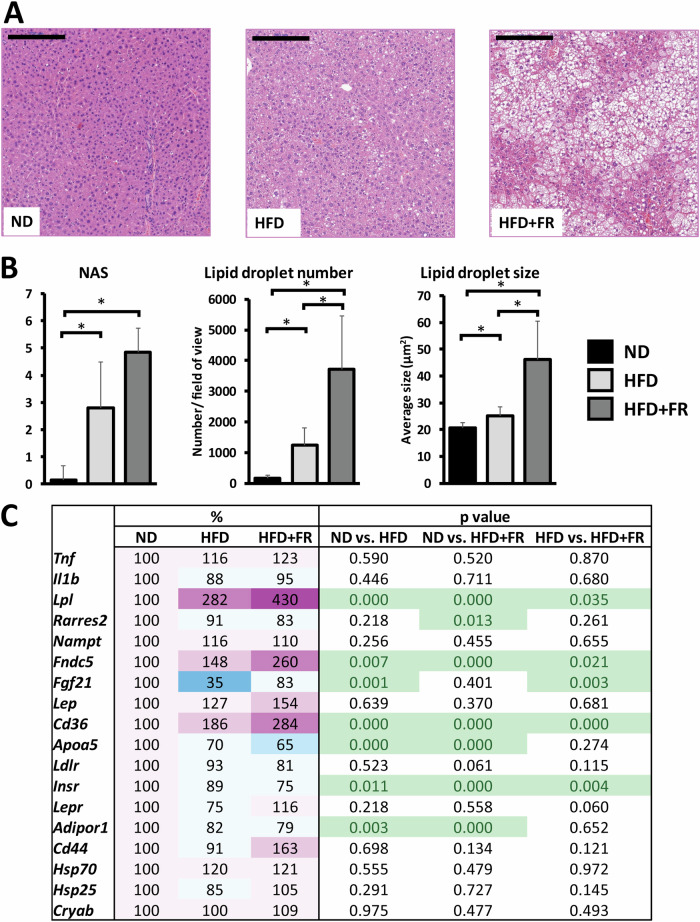
Effects of diets on hepatic lipid accumulation and hepatic gene expression. **A** Representative images of hematoxylin**–**eosin-stained liver sections of ND, HFD and HFD + FR animals. **B** Quantification of the results: left – NAFLD Activity Score (NAS), middle – number of lipid droplets, and right – size of the lipid droplets. **C** Heatmap of relative gene expression differences in the liver in response to diets. The relative expression of target genes in HFD or HFD + FR fed animals was compared to the expression level detected in control animals (results are given as a percentage of the ND group, where ND = 100%). Scale bar = 200 µm. Values are mean ± SD, *n* = 14-15/group, ^*^p < 0.05; ND normal diet, HFD high-fat diet, HFD + FR high-fat/high-fructose diet.

Obesity is usually accompanied by NAFLD. To study the severity of steatosis and inflammatory cell infiltration, we performed the histological observation of hematoxylin**–**eosin-stained liver sections (Fig. 2A). NAS was calculated based on the scores of steatosis, lobular inflammation and hepatocyte ballooning. Levels of steatosis and hepatocyte ballooning elevated in the liver of the HFD and HFD + FR animals, leading to significantly higher NAS in both obesity models compared with the control group (*p* < 0.05 for both obesity models) (Fig. 2B, left). On the other hand, we did not observe remarkable signs of inflammation in either the HFD or HFD + FR groups. In the liver of the healthy ND animals, we detected a minimal amount of lipid droplets (Fig. 2B, middle). A moderate increase was detected in the HFD group, whereas the number and size of lipid droplets dramatically elevated in the liver of HFD + FR animals leading to significant differences between the HFD and HFD + FR groups (*p* < 0.05 for both the number and size of lipid droplets) (Fig. 2B, middle and right).

To further study these processes, we analyzed the relative expression changes of genes involved in the regulation of hepatic lipid homeostasis and inflammation using qPCR (Fig. 2C). To reveal the effects of diets, we compared the values for the HFD and HFD + FR groups with the ND-fed controls (for all comparisons, ND = 100%). Expression level of the gene encoding lipoprotein lipase (*Lpl*) significantly increased in response to the diets, showing almost 3-fold increase in the HFD group and a more than 4-fold increase in the HFD + FR animals (*p* < 0.001 and *p* < 0.05, respectively). The mRNA level of fibroblast growth factor 21 (*Fgf21*) was significantly lower in the liver of HFD-fed animals (*p* < 0.01), however, its level only slightly decreased in the HFD + FR group compared to the controls. Gene expression of the fatty acid translocase *Cd36* did not reach two-fold increase in response to HFD alone (*p* < 0.001), but it showed a remarkable, almost 3-fold increase in the HFD + FR mice (*p* < 0.001). Similarly, expression level of *Fndc5*, encoding irisin, was only slightly elevated in the HFD group (*p* < 0.01), while it was significantly higher in the HFD + FR group (*p* < 0.001).

### Diet-induced inflammatory changes in adipose tissues and serum

Morphological changes in the interscapular brown adipose tissue (iBAT) were analyzed on hematoxylin-eosin stained sections. A high number of small lipid droplets were detected in the iBAT of the ND and HFD-fed animals (Supplementary Fig. S2A left, middle). However, in the case of the HFD + FR group, the size of the lipid droplets increased while their number decreased (Supplementary Fig. S2A right). Quantification of the results revealed significant differences between the ND and HFD + FR groups in terms of both the number and size of lipid droplets (*p* < 0.05) (Supplementary Fig. S2B left, right). Furthermore, there was a significant difference in droplet size between the HFD and HFD + FR groups (*p* < 0.05) (Supplementary Fig. S2B right).

**Fig. 3:**
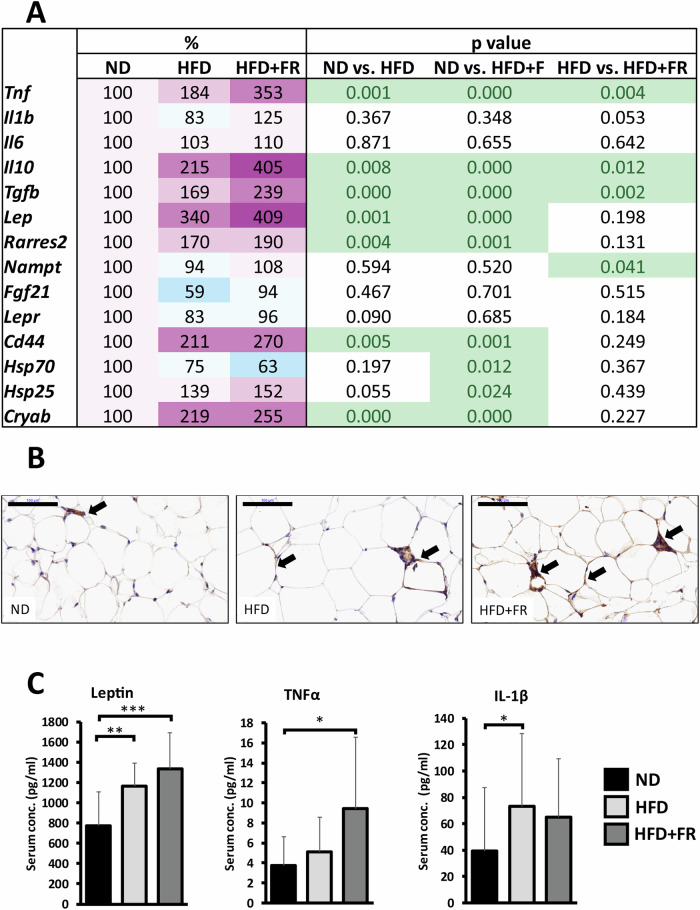
Diet-induced inflammatory changes in the visceral white adipose tissue and in the serum. **A** Heatmap of relative gene expression differences. The relative expression of target genes in HFD or HFD + FR animals was compared to the expression levels detected in control animals (results are given as a percentage of the ND group, where ND = 100%). **B** CD44 immunolabeling on vWAT sections of ND, HFD and HFD + FR-fed mice. Brown staining indicates CD44 immunoreactivity (black arrows). Nuclei were counterstained with hematoxylin. Scale bar: 100 µm. **C** Serum concentrations of leptin, TNFα, and IL-1β. Values are mean ± SD, *n* = 14-15/group, ^*^p < 0.05; ND normal diet, HFD high-fat diet, HFD + FR high-fat/high-fructose diet.

Next, we analyzed the gene expression levels of cytokines and adipokines in iBAT (Supplementary Fig. S2C) and visceral white adipose tissue (vWAT; Fig. 3A). Regarding the BAT, we found increased expression of the *Lep* gene, encoding leptin, in response to HFD (*p* < 0.05), and an even higher level in the HFD + FR group (*p* < 0.01) (Supplementary Fig. S2C). The mRNA level of another adipokine, *Fgf21* elevated significantly only in the HFD + FR animals (*p* < 0.01), and *Tnf* also showed a slight but statistically significant increase in the HFD + FR group compared to the controls (*p* < 0.01).

As expected, the vWAT expression level of the *Lep* gene was significantly higher in the HFD and HFD + FR groups than in the control animals (*p* < 0.01 and *p* < 0.001, respectively), while we could not detect significant differences between the two obesity models (Fig. 3A). Among the examined cytokines, the gene expression levels of *Il1b* and *Il6* were not affected, while those of *Tnf*, *Il10* and *Tgfb* were induced by both diets. In addition, all three markers showed higher expression in the HFD + FR group when compared with the HFD model (*Tnf*: *p* < 0.01; *Il10*: *p* < 0.05; *Tgfb*: *p* < 0.01; Fig. 3A). Gene expression level of the hyaluronate receptor *Cd44* increased to a similar extent due to both diets when compared with the control group (*p* < 0.01 and *p* < 0.01 in HFD and HFD + FR, respectively; Fig. 3A). The distribution of CD44 protein in the visceral adipose tissue was evaluated using immunohistochemical staining. The results demonstrated strong staining intensities in endothelial cells and mesenchymal stem cells, while adipocytes exhibited weaker staining (Fig. 3B). Among the studied heat shock protein genes, *Cryab*, encoding αB-crystallin, showed more than two-fold increase in response to the diets (*p* < 0.001 and *p* < 0.001 in HFD and HFD + FR, respectively). Moreover, we calculated the Pearson correlation coefficient to analyze the linear relationship between the expression levels of different genes. The results revealed strong positive linear correlation between the expression levels of *Lep* and *Cryab* as well as between *Lep* and *Hsp25* (Supplementary Fig. S3).

To confirm the results of the qPCR analysis, serum concentrations of adipokines and inflammatory cytokines were measured by multiplex immunoassays. Consistent with the qPCR results, the serum leptin concentration significantly elevated in the HFD and HFD + FR animals when compared with the controls (*p* < 0.01 and *p* < 0.001, respectively) without remarkable differences between the two diets (Fig. 3B, left). TNFα concentration was not raised by HFD alone, while it was significantly higher in the HFD + FR group than in the control animals (*p* < 0.05) (Fig. 3B, middle). In contrast, the increase in IL-1β concentration reached a statistically significant level only in the HFD group compared to the controls (*p* < 0.05) (Fig. 3B, right).

### Single-cell immunophenotyping

**Fig. 4:**
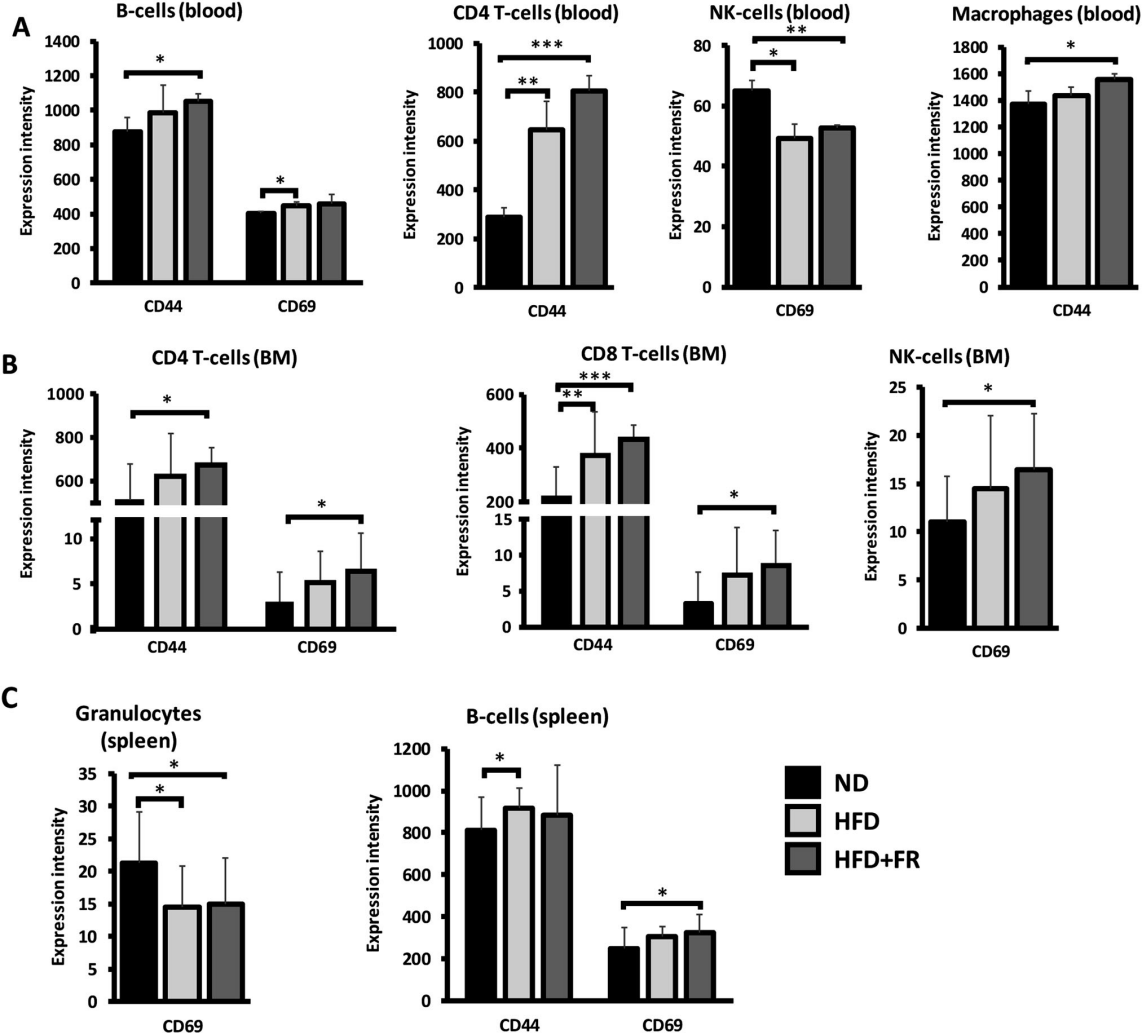
Mass cytometric immunophenotyping. **A** CD44 and CD69 expression intensity in B-cells, CD4 + T-cells, NK cells, and macrophages isolated from blood, **B** CD4+ and CD8 + T-cells, and NK cells isolated from bone marrow (BM) and **C** granulocytes and B-cells isolated from spleen. Values are mean ± SD, *n* = 14-15/group, ^*^p < 0.05, ^**^p < 0.01, ^***^p < 0.001; ND normal diet, HFD high-fat diet, HFD + FR high-fat/high-fructose diet.

Finally, we performed immunophenotyping of cells isolated from blood, bone marrow and spleen. In response to HFD + FR, the analysis revealed increased expression of the CD44 cell surface glycoprotein on B-cells (*p* < 0.05), CD4 + T-cells (*p* < 0.05), and macrophages (*p* < 0.05) of the blood (Fig. 4A) as well as on CD4+ and CD8 + T-cells of the bone marrow (*p* < 0.001 and *p* < 0.05, respectively; Fig. 4B). The CD69 type II C-lectin receptor increased on B-cells of the blood (*p* < 0.05) upon HFD diet (Fig. 4A), while HFD + FR induced elevated CD69 expression on CD4+ and CD8 + T-cells, on NK cells of the bone marrow (*p* < 0.05) (Fig. 4B), and on splenic B-cells (*p* < 0.05) (Fig. 4C). Interestingly, CD69 decreased (*p* < 0.05) on the surface of circulating NK cells and splenic granulocytes (*p* < 0.05) after both HFD and HFD + FR (Fig. 4).

## Discussion

Obesity is a multifactorial disorder characterized by chronic, low-grade inflammation throughout the body, especially in insulin-responsive organs, such as adipose tissues, liver, muscle or the pancreas. This chronic inflammatory state is caused by activated immune cells, mainly macrophages accumulating in these tissues, leading to an increased expression of pro-inflammatory cytokines [22]. In turn, many of the diseases associated with obesity, such as diabetes, NAFLD, or even cancer can be traced back to this low-grade inflammation. However, the exact mechanism and the causality of these processes are not yet fully understood.

In this study, we used two diet-induced mouse models, HFD and HFD + FR, to investigate the pathological changes and inflammatory processes induced by obesity at systemic level and in individual organs. HFD treatment is widely used to model obesity, hyperlipidemia and hyperglycemia in mice [9]. On the other hand, dietary sugars have been shown to influence the effects of HFD [23]. Fructose metabolism is largely different from that of glucose. For example, fructose does not trigger insulin release, and the main site of fructose metabolism is the liver [24]. Moreover, it has potent lipogenic effects, functioning as a substrate for fatty acid synthesis and activating the associated enzymes [23]. Consequently, excessive fructose intake strongly promotes the development of NAFLD and hepatic IR [25]. On the other hand, fructose-feeding alone did not lead to increased serum cholesterol level and seemed to induce lipid accumulation only in the liver [26]. Therefore, the combination of high-fat feeding with fructose supplementation is probably more effective to initiate metabolic syndrome in animal models.

Accordingly, we found remarkable differences between the two diet models regarding certain obesity-related pathological changes. Most interestingly, although the 5-month-long HFD resulted in significant weight gain and a mild NAFLD, it did not induce IR. In contrast, mice receiving fructose-supplemented water in addition to HFD showed higher peak glucose concentrations in the OGTT. This indicates that HFD + FR has a more significant negative effect on glucose metabolism than HFD alone, leading to the development of IR. In addition, compared to HFD, HFD + FR resulted in even higher weight gain, more severe symptoms of hepatic steatosis and lipid accumulation in the BAT. As opposed to WAT, the main function of BAT is not energy storage but non-shivering thermogenesis, therefore brown adipocytes contain high number of small lipid droplets. However, in response to excessive calorie intake, lipid accumulation can also be observed in the BAT, resulting in a structure that resembles WAT with adipocytes containing fewer but larger lipid droplets [11]. Such morphological changes of the BAT were observed only in the HFD + FR group. On the other hand, we did not find differences in serum lipid parameters between the two models. While the serum triglyceride level did not increase significantly by either diet, the LDL- and HDL-cholesterol levels elevated in similar extents in response to both diets. This suggests that serum hyperlipidemia is mainly induced by dietary fats, and fructose consumption has no significant additional effect, at least in the studied mouse model.

Because fructose supplementation exacerbated metabolic disturbances, a more severe inflammation was expected in these animals. Therefore, we examined several characteristics of inflammation, such as cytokine expression and immune cell activation in different organs and in the blood. NAFLD is a spectrum of changes caused by triglyceride accumulation from early-stage steatosis to chronic inflammation (steatohepatitis), which can finally lead to fibrosis and cirrhosis [27, 28]. In our experiments, we found that HFD alone caused mild steatosis, but more severe symptoms were observed when it was combined with fructose supplementation. The considerably higher level of NAS was a result of the more advanced lipid accumulation and hepatocyte ballooning. Consistent with this, several genes that are involved in the regulation of lipid accumulation, such as lipoprotein lipase, the fatty acid translocase *Cd36* or the gene encoding the hormone-like molecule irisin, were induced in response to HFD alone, and showed an even higher increase in the HFD + FR animals. On the other hand, we could not detect histopathological signs of inflammation or increased hepatic cytokine expression. Therefore, these results show that fructose supplementation worsened lipid accumulation but, at this stage of the disease, did not cause inflammation in the liver.

According to our current knowledge, the main contributors to obesity-induced systemic inflammation are the cytokines and other hormone-like molecules released by the WAT [29]. Indeed, the gene expression level of *Lep* was increased in parallel with higher body weight in our models, although we could not detect significant differences between the two diets. Three cytokines (*Tnf*, *Il10* and *Tgfb*) showed elevated expression in response to HFD, which increased further upon fructose supplementation. On the other hand, the mRNA levels of *Il6* and *Il1b* did not elevate in the WAT of animals with obesity. In recent years, increasing evidence supports that the molecular chaperone heat shock proteins are involved in the regulation of inflammation and certain metabolic disturbances [30–32]. In the current study, we also observed significant increase in the small heat shock protein *Cryab* (and to a smaller extent in *Hsp25*) mRNA level in the WAT of the HFD and HFD + FR animals, while the level of *Hsp70* rather decreased. In addition, we registered strong positive linear correlation between the expression levels of *Lep* and *Cryab* or *Hsp25*. These results suggest a specific role of small heat shock proteins in the regulation of obesity-related metabolic alterations, which is in agreement with previous research revealing that αB-crystallin functions as an adipokine [33] and might be involved in the pathogenesis of diet-induced diabetes [34].

Serum concentrations of leptin and TNFα were consistent with the mRNA levels measured in the adipose tissues. In contrast, *Il1b* gene expression was not induced by the applied diets either in the WAT or in the BAT but the protein was present in higher concentrations in the blood of animals with obesity compared with the control group, suggesting other potential sources of circulating Il-1β. According to some assumptions, certain cytokines secreted into the blood are responsible for the development of IR. Both TNFα and Il-1β were found to be able to influence insulin signaling, although it was also shown that circulating level of TNFα is lower than the effective concentration even in patients with obesity [22]. In our experiments, serum TNFα concentration was higher in the HFD + FR animals in parallel with the higher level of IR compared with the HFD group. In contrast, serum Il-1β concentration was not different between the two diet groups, which implies that Il-1β alone can not be responsible for the development of IR.

Because increased serum concentration of pro-inflammatory cytokines indicates a chronic systemic inflammation, we analyzed the systemic immune changes by single-cell phenotyping of all major immune cell populations in the blood, bone marrow and spleen. The most prominent changes were found in the surface expression levels of CD69 and CD44. CD69 is an early marker of immune cell activation and an important regulator of immune responses [35]. Indeed, in the animals with obesity we found its increased surface expression on T-cells and NK cells in the bone marrow samples, and on B-cells of the blood and spleen. On the other hand, circulating NK cells showed decreased surface expression of CD69 in response to obesity. CD69 on NK cells appears to play a crucial role in initiating tumor cell lysis [36], therefore its reduced expression may impair the anti-tumor response. Indeed, increasing evidence suggests that obesity-related alterations in NK cell physiology and function may influence tumor development [37]. The surface expression of CD44 was also significantly increased in response to the applied diets in various cell types, such as B-cells, macrophages or CD4+ and CD8 + T-cells. CD44 is a multifunctional cell surface glycoprotein, a receptor for hyaluronan and osteopontin. It participates in the activation and proliferation of T-cells and NK cells, therefore these results also confirm the development of systemic inflammation in the animals with obesity. However, CD44 is expressed by other cell types as well, such as hepatocytes or adipocytes, which has an important role in the diet-induced adipose inflammation [38]. Indeed, our qPCR results showed that *Cd44* expression in the vWAT displayed a robust elevation in accordance with the higher level of cytokine expression. Previously, an increased frequency of CD44 + T-cells was observed in subcutaneous adipose tissue of HFD-fed mice [39] which can contribute to the increased *Cd44* mRNA level in the vWAT of our mice as well. However, in line with the findings of previous research [40, 41], the results of the CD44 immunostaining indicated that endothelial cells, mesenchymal stem cells, and to a lesser extent, adipocytes may also be important sites of *Cd44* expression. Moreover, it should be mentioned that CD44 is also involved in the regulation of cell adhesion and migration, and it is an important regulator of cancer cell progression and metastasis [42]. We can therefore suppose that the increased CD44 expression in the WAT might be another important link between obesity-induced inflammation and the higher risk of cancer. Interestingly, we did not find significant differences in the surface expression of CD69 and CD44 between the two obesity models, suggesting that even mild obesity can induce these immunophenotypic changes.

It is important to acknowledge the limitations of the present study, in particular the fact that only males were included. In our previous studies we found that females are more resistant to the development of diet-induced obesity and related disorders, such as NAFLD [11, 12]. Because here our major aim was to investigate obesity-related inflammation, we decided to use only male animals, which have a higher degree of obesity. Moreover, this allowed us to increase the number of animals per groups.

In conclusion, although HFD alone is suitable for examining certain obesity-related parameters, its supplementation with fructose led to a better model of Western-diet. HFD + FR resulted in a more substantial increase in weight and in the level of hepatic steatosis, and only these animals showed IR. Accordingly, fructose supplementation resulted in increased serum TNFα concentration and expression of specific cytokines in the WAT (and BAT), indicating enhanced systemic inflammation. However, despite these differences, both models showed immunophenotypic alterations that may be linked to an increased risk of obesity-related cancer. On the other hand, the extent of inflammation did not correlate with all symptoms of metabolic syndrome as neither serum triglyceride increased nor steatohepatitis was detected in either obesity models.

## Supplementary information

Supplementary Methods and Figure legends

Figure S1

Figure S2

Figure S3

## Data Availability

The datasets used and/or analyzed during the current study are available from the corresponding author on reasonable request.
