# Supplementary material for: Characterization of obesity-related diseases and inflammation using single cell immunophenotyping in two different diet-induced obesity models
Source: Int J Obes (Lond). 2024 Jul 14;48(11):1568–76. doi: 10.1038/s41366-024-01584-6 (PMC11502477; doi:10.1038/s41366-024-01584-6)
Supplement: Supplementary file 1 — Supplementary Methods and Figure legends [file 41366_2024_1584_MOESM1_ESM.docx]

**Supplementary materials**

**Supplementary Materials and Methods**

**Serum triglyceride, total cholesterol, LDL-cholesterol and HDL-cholesterol levels**

Serum triglyceride, total, LDL- and HDL-cholesterol levels were determined using commercially available enzymatic colorimetric assay kits (Diagnosticum Ltd., Budapest, Hungary) according to the manufacturer’s instructions. Fasting blood samples were collected through cardiac puncture under terminal anesthesia. After clot formation, samples were centrifuged at 4 °C, 1000 × g for 10 min, then serum was removed and stored at −80 °C until use. Each serum sample was measured in triplicate. Test accuracy was monitored using Standard Lipid Controls (Diagnosticum Ltd., Budapest, Hungary). The absorbance of the produced purple/blue color product was measured at 505/596 nm, respectively, using a microplate reader (Multiskan FC, ThermoScientific, United States). Values were used to calculate the serum triglyceride, total, LDL- and HDL-cholesterol concentrations, expressed in mmol/l.

**RNA isolation and quantitative real-time polymerase chain reaction (qPCR)**

Total RNA was isolated using RNeasy Fibrous Tissue Mini Kit (Qiagen, Hilden, Germany). High Capacity cDNA Reverse Transcription Kit (Thermo Fisher Scientific, Waltham, Massachusetts, USA) was used to convert RNA samples to cDNA. Each reaction mixture contained 1 µg RNA (15 µl), 1.5 µl MultiScribe Reverse Transcriptase, 3 µl primer, 1.2 µl dNTP, 3 µl buffer, 6.3 µl RNase-free water. Parameters for the reverse transcription program were the following: incubation at 25 °C for 10 min, reverse transcription at 37 °C for 2 h, and inactivation at 85 °C for 5 min (using BioRad T100 Thermal Cycler, Hercules, CA, USA). The cDNA product was finally diluted at 1:20 and used as a qPCR reaction template. For the qPCR reaction, 10 µl cDNA, 1 µl (250 nM final) primer mix (forward+reverse), and 10 µl Power SYBR Green PCR Master Mix 2x (Thermo Fisher Scientific, Waltham, Massachusetts, USA) were mixed in a total volume of 20 µl. The reaction was performed on a RotorGene 3000 instrument (Qiagen, Hilden, Germany) with the following settings: heat activation at 95 °C for 10 min followed by 40 cycles of denaturation at 95 °C for 15 s, annealing at 60 °C for 60 s. Melting curve analysis was performed between 50–95°C to verify the specificity of the amplification. Primer sequences used in qPCR reactions are listed in Table S2. The mouse Gapdh gene served as an internal control for normalization. Relative gene expression levels were calculated using the ΔΔCt method.

**Cell isolation**

Blood collection was performed using 50 µl of 30 mg/ml EDTA for 1 ml of blood in a 1 ml syringe. Spleen or lymph nodes were homogenized using a 100 µm cell strainer (Merck) and the plunger of a 2 ml syringe, washed with PBS (Merck). Bone marrow was isolated by 1 ml PBS washing of the femur and tibia following the cut of the epiphysis. To lyse erythrocytes of blood, spleen, and bone marrow samples, 5 ml of ACK lysis buffer (ACK: 0.15 M NH_4_Cl, 10 mM KHCO_3_, 0.1 mM Na_2_EDTA, pH 7.3; Merck, Darmstadt, Germany) was applied at room temperature for 5 min. Samples were washed twice with 10 ml PBS, and subsequently cell counting and viability check were performed with Trypan Blue exclusion. Cryopreservation of cells was carried out in stocks of 4 × 10^6^ cells in 1 ml FBS (Capricorn Scientific, Ebsdorfergrund, Germany) supplemented with DMSO (Merk) at 1:10 ratio in cryotubes (Greiner Bio-One) in liquid nitrogen (Messer, Bad Soden, Germany).

**Cell preparation for mass cytometry (CyTOF)**

Cyrotubes were thawed in a 37 °C water bath for 2 min and cells were transferred into 14 ml RPMI supplemented with 10% FBS (Capricorn Scientific), and centrifuged at 350 *g* for 6 min at RT. Cells were washed again with 10 ml RPMI 10% FBS, counted, and viability was determined with Trypan Blue exclusion. Cells were plated in 96-well repellent plates (2.5 × 10^6^ cells in 200 ul RPMI 10% FBS/well). After 2 h, cells were stimulated with 10 ng/ml PMA, 1 µg/ml ionomycin and 5 µg/ml Brefldin A, and left overnight at 37 °C. Cells were collected and washed twice with Maxpar Cell Staining Buffer (MCSB; Fluidigm, South San Francisco,USA).

**Antibody staining**

Viability was determined by cisplatin (5 µM ^195^Pt, Fluidigm) staining for 3 min on ice in 500 µl PBS. Samples were diluted with 3 ml Maxpar Cell Staining Buffer (MCSB, Fluidigm) and centrifuged at 350 *g* for 5 min. Cells were resuspended in 50 µl MCSB supplemented with TrueStain FcX™ PLUS FC receptor blocking solution (Biolegend, San Diego, USA) at 1:100 (v/v), and incubated at RT for 10 min. Without a washing step, samples were barcoded by adding 50 µl of different metal-tagged (89Y, 147Sm, 175Lu) CD45 antibodies (clone:30-F11; Fluidigm) at a final concentration of 1:100 (v/v) per antibody and incubated at 4 °C for 30 min. Following barcoding, 3 samples were pooled for the subsequent antibody staining. First, cells were stained with the commercially available Maxpar Mouse Spleen/Lymph Node Phenotyping panel (Cat.num.:201306, Fluidigm), incubated at 4 °C for 30 min, washed twice with 2 ml MCSB. Fixation was performed with 1 ml Maxpar Fix I buffer (5x) diluted in PBS, incubated at RT for 30 min and washed twice with Maxpar PermS Buffer, centrifuged at 800 *g* for 5 min. Cells were stained with the Maxpar Mouse Intracellular Cytokine I panel kit (Cat.num.:201310, Fluidigm), incubated at RT for 30 min, washed twice with MCSB, and prefixed with 1 ml Pierce™ formaldehyde (Thermo Scientific) solution diluted in PBS to 1.6%, and incubated at RT for 10 min. Stained and pre-fixed cells were centrifuged at 800 *g* at RT for 6 min and resuspended in 800 µl Fix & Perm solution (Fluidigm) supplemented with Ir^191^-Ir^193^ DNA intercalator (Fluidigm) at 1:1000 ratio (v/v) for overnight incubation.

**Mass cytometry by time of flight (CyTOF) measurement**

Samples were washed twice with MCSB and once with PBS prior filtering through 30 μm Celltrics (Sysmex, Kobe, Japan) gravity filter (Sysmex, Görlitz, Germany), and the cell concentration was adjusted to 7x10^5^/ml in Maxpar Cell Acquisition Solution (Fluidigm). Finally, EQ four element calibration beads (Fluidigm) were added at 1:10 ratio (v/v), and acquisitions were performed on a properly tuned Helios mass cytometer (CyTOF, Fluidigm) by collecting 3 × 10^5^ events per individual PBMC. The generated flow cytometry standard (FCS) files were randomized and normalized with the default setting of the internal FCS-processing unit of the CyTOF Software (Fluidgm, version:7.0.8493). Manual gating was used in Cytobank (Beckman Coulter) to gate the populations and determine marker expression intensities.

**Luminex MAGPIX**

After withdrawal of 1 ml blood, samples were centrifuged at 350 *g* for 10 min at 4 °C, plasma fractions were stored at −80 °C in aliquots before running the assay. Luminex xMAP (MAGPIX®) technology was used to determine the protein concentrations of 20 distinct cytokines/chemokines (IL-10, IL-1b, IL-2, IP-10, IL-4, IL-6, PD-L1, IL-23, INF-g, IL-12p70, CXCL1, RANTES(CCL5), TNF-a, TIM-3, MCP-1(CCL2), IL-17A, MIP-2a(CXCL2), CXCL5, Leptin, CTLA4). The ProcartaPlex™ Immunoassay (ThermoFisher Scientific, Waltham, USA) was perfomed according to the instructions of the manufacturer. Briefly, all samples were thawed and were tested in a blind fashion and in duplicate. 40 μl volume of each sample, 25 μl volume of standard, and universal assay buffer was added to a 96-well plate (provided with the kit) containing 50 μl of capture antibody-coated, fluorescent-coded beads. Biotinylated detection antibody mixture and streptavidin-PE were added to the plate after the appropriate incubation period. After the last washing step, 120 μl reading buffer was added to the wells, the plate was incubated for an additional 5 min, and read on the Luminex MAGPIX® instrument. Luminex xPonent 4.2 software was used for data acquisition. Five-PL regression curves were generated to plot the standard curves for all analytes by the Analyst 5.1 (Merck) software calculating with bead median fluorescence intensity values. Data were pooled from two independent measurements and plotted in GraphPad Prism v8 (Dotmatics, Boston, USA).

**Legendplex**

After withdrawal of 1 ml blood, samples were centrifuged at 350 *g* for 10 min at 4 °C, plasma fractions were stored at −80 °C in aliquots before running the assay. Legendplex technology, a multiplex bead-based immunoassay was used to determine the protein concentrations of 13 distinct cytokines/chemokines (IL-23, IL-1a, IFN-g, TNF-a, MCP-1, IL12p70, IL-1b, IL-10, IL-6, IL-27, IL-17A, IFN-b, GM-CSF) of the Mouse Inflammation panel (Cat num.:740446, Biolegend) according to the instructions of the manufacturer. Briefly, plasma samples were incubated with the premixed capture beads, and following washing, detection antibodies were added. After washing, streptavidin-PE was finally added. A standard curve was generated by the application of the commercial cytokine standards of the kit. Minimal detectable concentrations were as follows: IL-23: 4.2 pg/ml; IL-1a: 1.3 pg/ml; IFN-g: 0.8 pg/ml; TNF-a: 1.9 pg/ml; MCP-1: 1.7 pg/ml; IL12p70: 0.7 pg/ml; IL-1b: 2.8 pg/ml; IL-10: 2.1 pg/ml; IL-6: 0.9 pg/ml; IL-27: 9.8 pg/ml; IL-17A: 1.8 pg/ml; IFN-b: 4 pg/ml; and GM-CSF: 1.9 pg/ml. Samples were acquired on a Cytoflex S FACS (Beckman Coulter) using the APC and PE channels. Evaluation was performed in CytExpert 4.0 (Beckman Coultrer) and Microsoft Excel by fitting the median values to the strandard curves.

**Supplementary figure legends**

**Fig. S1.** Weight of the **A)** liver, **B)** brown adipose tissue (BAT), **C)** spleen, **D)** kidney, **E)** heart and **F)** brain. Values are mean ± SD, n = 14-15/group, ^*^p < 0.05; ND = normal diet, HFD = high-fat diet, HFD+FR = high-fat/high-fructose diet.

**Fig. S2.** Effects of diets on morphological and gene expression changes in the brown adipose tissue. **A)** Representative images of hematoxylin**–**eosin-stained sections of control, HFD and HFD+FR animals. **B)** Quantification of the results: number and size of lipid droplets. **C)** Heatmap of relative gene expression differences in response to diets. The relative expression of target genes in HFD or HFD+FR fed animals was compared to the expression levels detected in control animals (results are given as a percentage of the ND group, where ND = 100%). Scale bar = 200µm. Values are mean ± SD, n = 14-15/group, ^*^p < 0.05; ND = normal diet, HFD = high-fat diet, HFD+FR = high-fat/high-fructose diet.

**Fig. S3.** Pearson correlation coefficient (r) and p-value (p) between the gene expression levels of **A)** *Lep* (leptin) and *Cryab* (αB-crystallin) and **B)** *Lep* and *Hsp25*.

**Supplementary tables**

|  | Normal Diet | High-fat Diet |
| --- | --- | --- |
| Moisture (%) | 10 | 10 |
| Crude Oil (%) | 4.75 | 21.65 |
| Crude Protein (%) | 19.11 | 19.31 |
| Crude Fibre (%) | 3.85 | 3.65 |
| Ash (%) | 6.97 | 4.97 |
| NFE (%) | 55.32 | 39.34 |
| **Table S1**. Composition of normal and high-fat diet. High-fat Diet contains 0.15% of supplementary cholesterol (giving a total of 2% cholesterol). | | |

|  | **gene** | **forward primer** | **reverse primer** |
| --- | --- | --- | --- |
| **1** | ***Gapdh*** | GGGTTCCTATAAATACGGACTGC | CCATTTTGTCTACGGGACGA |
| **2** | ***Lep*** | GTGGCTTTGGTCCTATCTGTC | CGTGTGTGAAATGTCATTGATCC |
| **3** | ***Fgf21*** | GTGTCAAAGCCTCTAGGTTTCTT | GGTACACATTGTAACCGTCCTC |
| **4** | ***Nampt*** | GCAGAAGCCGAGTTCAACATC | TTTTCACGGCATTCAAAGTAGGA |
| **5** | ***Rarres2*** | GCCTGGCCTGCATTAAAATGG | CTTGCTTCAGAATTGGGCAGT |
| **6** | ***Tnf*** | CCCTCACACTCAGATCATCTTCT | GCTACGACGTGGGCTACAG |
| **7** | ***Il1b*** | GCAACTGTTCCTGAACTCAACT | ATCTTTTGGGGTCCGTCAACT |
| **8** | ***Tgfb1*** | CTCCCGTGGCTTCTAGTGC | GCCTTAGTTTGGACAGGATCTG |
| **9** | ***Il10*** | CAGAGCCACATGCTCCTAGA | TGTCCAGCTGGTCCTTTGTT |
| **10** | ***Lepr*** | AGCTAGGTGTAAACTGGGACA | GCAGAGGCGAATCATCTATGAC |
| **11** | ***Cd36*** | TTGAAAAGTCTCGGACATTGAG | TCAGATCCGAACACAGCGTA |
| **12** | ***Insr*** | TCAAGACCAGACCCGAAGATT | TCTCGAAGATAACCAGGGCATAG |
| **13** | ***Ucp1*** | AGGCTTCCAGTACCATTAGGT | CTGAGTGAGGCAAAGCTGATTT |
| **14** | ***Cox8b*** | TGTGGGGATCTCAGCCATAGT | AGTGGGCTAAGACCCATCCTG |
| **15** | ***Cidea*** | TGACATTCATGGGATTGCAGAC | GGCCAGTTGTGATGACTAAGAC |
| **16** | ***ApoA5*** | GTGCTGCTGTCCCAACCTA | CTGAGTGTAATGCCCCTGAGT |
| **17** | ***AdipoR1*** | TGGTCTTCGGGATGTTCTTC | CCCTGAATAGTCCAGTTTGGAA |
| **18** | ***Ldlr*** | GTGGCTGAAAACCTCTTGTCC | CACACCAGTTCACCCCTCTA |
| **19** | ***Hsp25*** | ATCCCCTGAGGGCACACTTA | GGAATGGTGATCTCCGCTGAC |
| **20** | ***Fndc5*** | TTGCCATCTCTCAGCAGAAGA | GGCCTGCACATGGACGATA |
| **21** | ***Il6*** | GCTACCAAACTGGATATAATCAGGA | CCAGGTAGCTATGGTACTCCAGAA |
| **22** | ***Cryab*** | GTTCTTCGGAGAGCACCTGTT | GAGAGTCCGGTGTCAATCCAG |
| **23** | ***Hsp70*** | GAGATCGACTCTCTGTTCGAGG | GCCCGTTGAAGAAGTCCTG |
| **24** | ***Actb*** | CTAAGGCCAACCGTGAAAAG | ACCAGAGGCATACAGGGACA |
| **25** | ***Cd44*** | CACCTTGGCCACCACTCCTA | TCCGTTCTGAAACCACGTCT |
| **26** | ***Lpl*** | CATGGATGGACGGTAACGGG | TTCTCTCTTGTACAGGGCGG |

**Table S2**. Primer sequences used fo qPCR
