# Supplementary figures and images for: Characterization of obesity-related diseases and inflammation using single cell immunophenotyping in two different diet-induced obesity models
Source: Int J Obes (Lond). 2024 Jul 14;48(11):1568–76. doi: 10.1038/s41366-024-01584-6 (PMC11502477; doi:10.1038/s41366-024-01584-6)

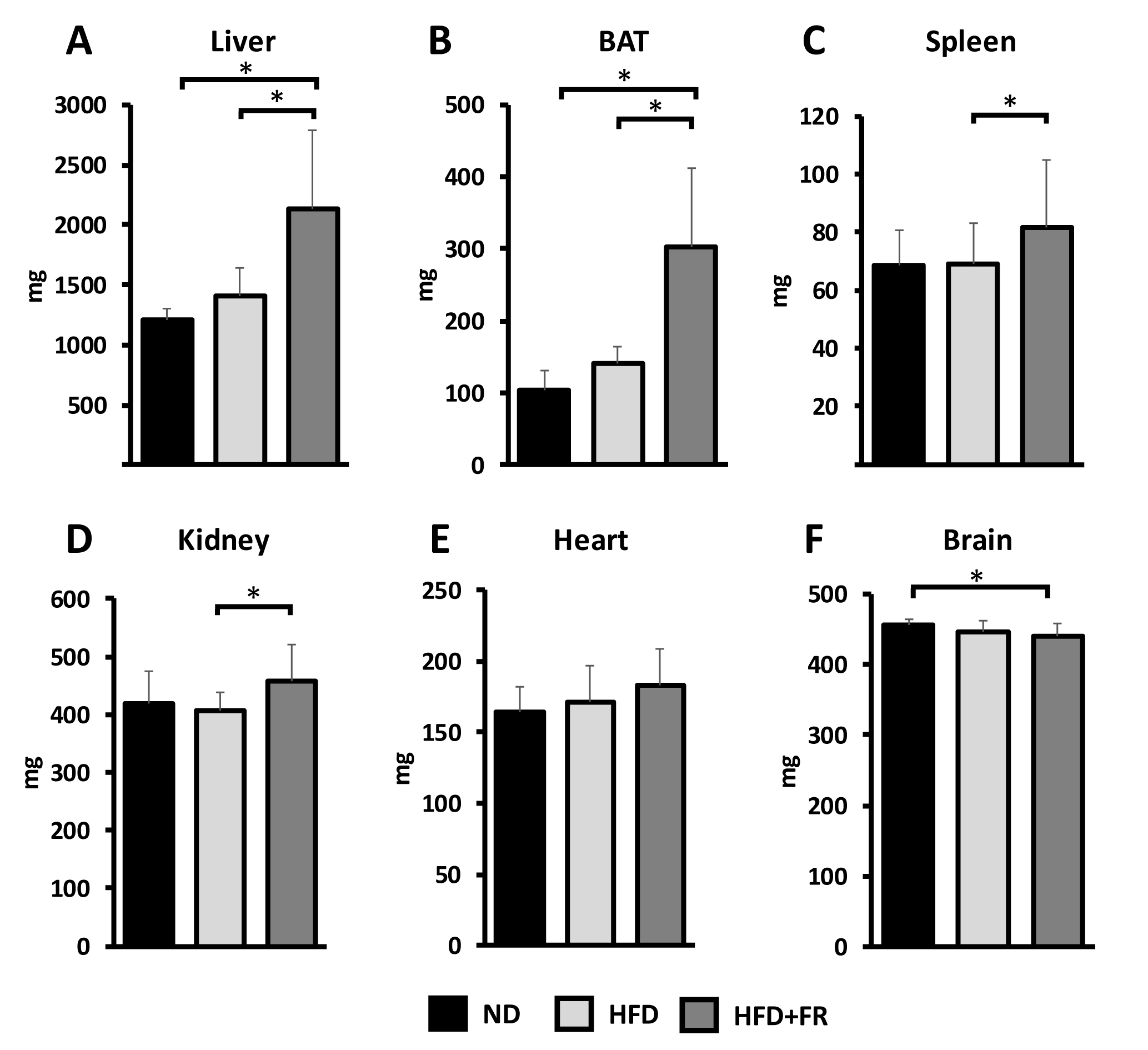

Supplement: Supplementary file 2 — Figure S1 [file 41366_2024_1584_MOESM2_ESM.jpg]

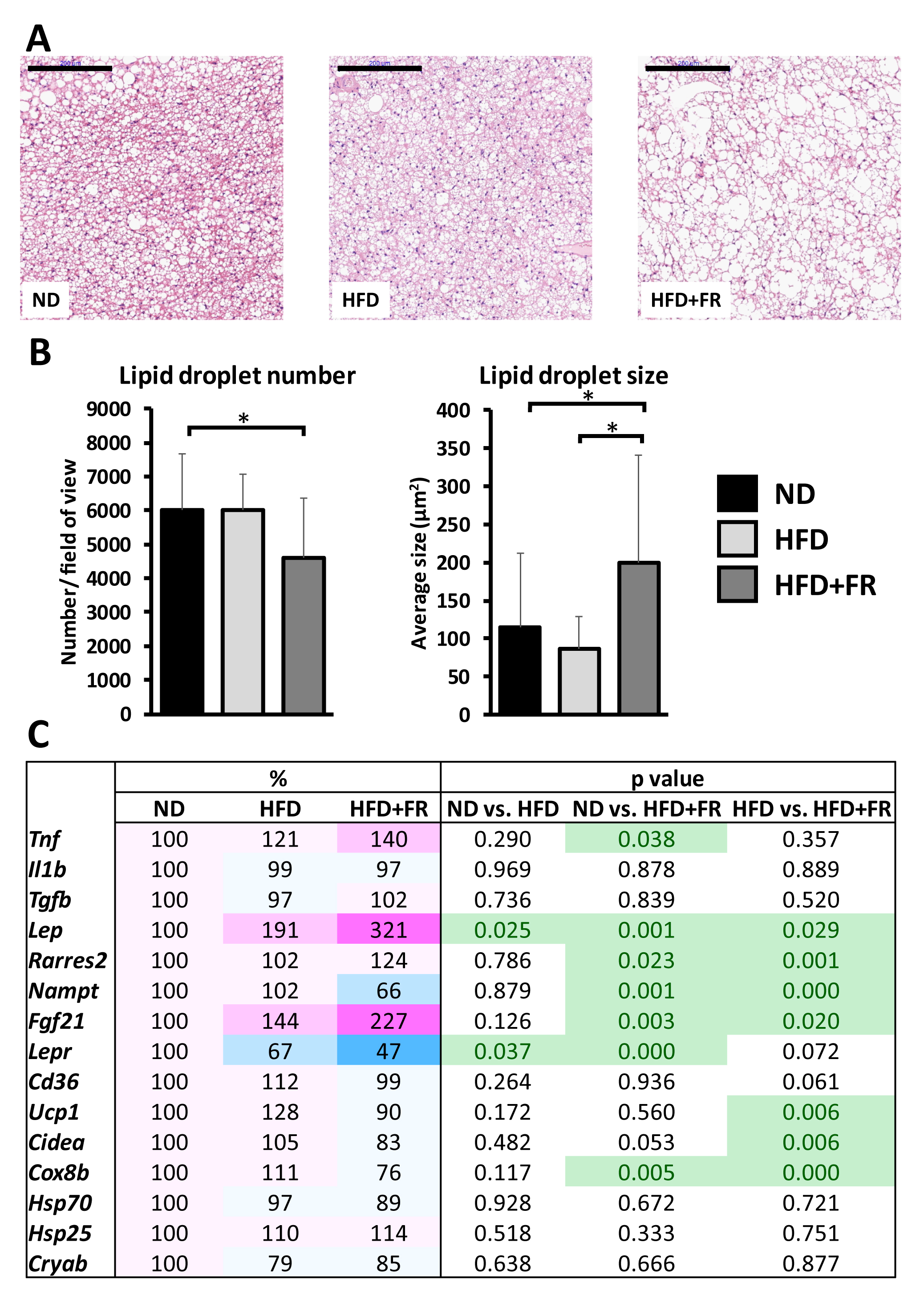

Supplement: Supplementary file 3 — Figure S2 [file 41366_2024_1584_MOESM3_ESM.jpg]

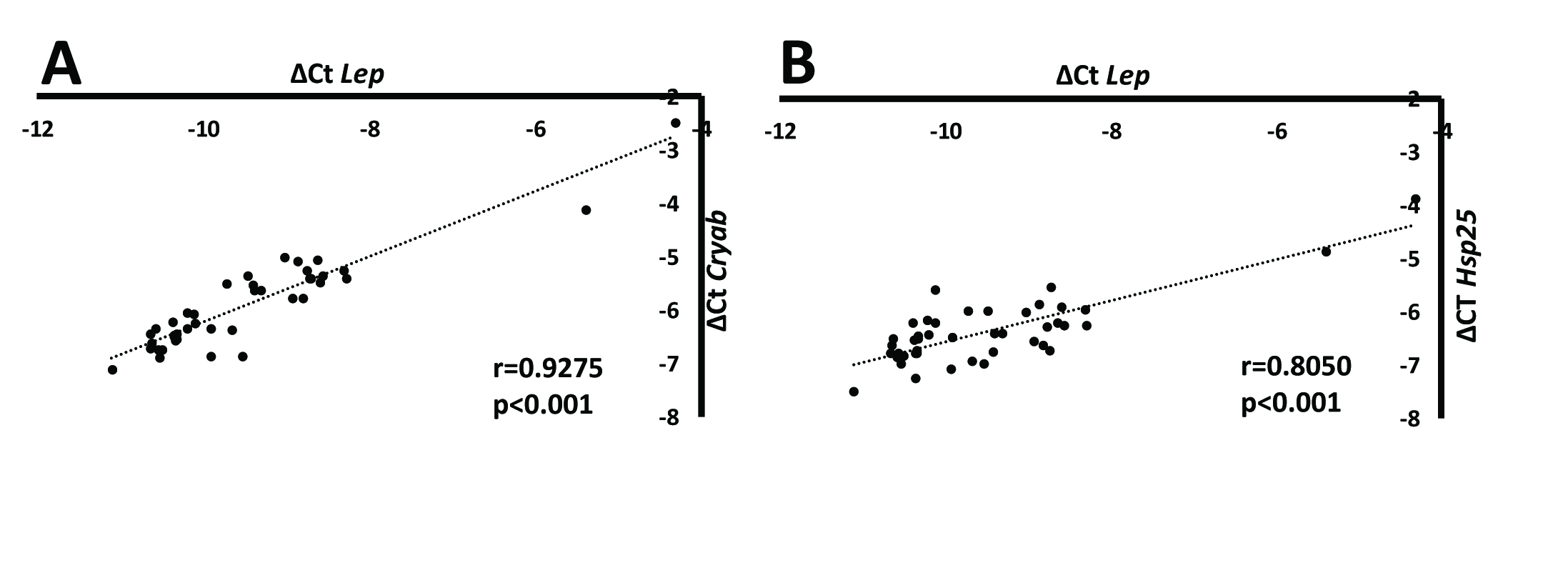

Supplement: Supplementary file 4 — Figure S3 [file 41366_2024_1584_MOESM4_ESM.jpg]
